# Distinct patterns of biomarker expression for atypical intraductal proliferations in prostate cancer

**DOI:** 10.1007/s00428-023-03643-1

**Published:** 2023-09-14

**Authors:** Carmela Martini, Jessica M. Logan, Alexandra Sorvina, Sarita Prabhakaran, Benjamin S Y. Ung, Ian R. D. Johnson, Shane M. Hickey, Robert D. Brooks, kConFab Consortium, Maria C. Caruso, Sonja Klebe, Litsa Karageorgos, John J. O’Leary, Brett Delahunt, Hemamali Samaratunga, Douglas A Brooks

**Affiliations:** 1 Clinical and Health Sciences, University of South Australia, Adelaide, Australia; 2 Department of Anatomical Pathology, College of Medicine and Public Health, Flinders University, Adelaide, SA Australia; 3 Department of Surgical Pathology, SA Pathology at Flinders Medical Centre, Adelaide, Australia; 4 Department of Histopathology, Trinity College Dublin, Dublin, Ireland; 5 Malaghan Institute for Medical Research, Wellington, New Zealand; 6 Aquesta Uropathology and the University of Queensland, Brisbane, Qld Australia

**Keywords:** High-grade prostatic intraepithelial neoplasia, Atypical intraductal proliferation, Immunohistochemistry, Biomarkers, Diagnosis, Prostatic adenocarcinoma

## Abstract

High-grade prostatic intraepithelial neoplasia (HGPIN) is a well-characterised precursor lesion in prostate cancer. The term atypical intraductal proliferations (AIP) describes lesions with features that are far too atypical to be considered HGPIN, yet insufficient to be diagnosed as intraductal carcinoma of the prostate (IDCP). Here, a panel of biomarkers was assessed to provide insights into the biological relationship between IDCP, HGPIN, and AIP and their relevance to current clinicopathological recommendations. Tissue samples from 86 patients with prostate cancer were assessed by routine haematoxylin and eosin staining and immunohistochemistry (IHC) with a biomarker panel (Appl1/Sortilin/Syndecan-1) and a PIN4 cocktail (34βE12+P63/P504S). Appl1 strongly labelled atypical secretory cells, effectively visualising intraductal lesions. Sortilin labelling was moderate-to-strong in > 70% of cases, while Syndecan-1 was moderate-to-strong in micropapillary HGPIN/AIP lesions (83% cases) *versus* flat/tufting HGPIN (≤ 20% cases). Distinct biomarker labelling patterns for atypical intraductal lesions of the prostate were observed, including early atypical changes (flat/tufting HGPIN) and more advanced atypical changes (micropapillary HGPIN/AIP). Furthermore, the biomarker panel may be used as a tool to overcome the diagnostic uncertainty surrounding AIP by supporting a definitive diagnosis of IDCP for such lesions displaying the same biomarker pattern as cribriform IDCP.

## Background

High-grade prostatic intraepithelial neoplasia (HGPIN) comprises three morphological patterns: flat, tufting, and micropapillary (MP) [1]. Atypical intraductal cribriform proliferations with cytological features of malignancy are no longer considered a HGPIN pattern, but now classified as IDCP (loose/dense), with patients displaying similar clinical behaviour to other IDCP patterns [2, 3]. In instances where atypical intraductal proliferative lesions are architecturally and/or cytologically more complex than HGPIN, but fall short of meeting the criteria of IDCP, the term atypical intraductal proliferations (AIP) is assigned to communicate diagnostic uncertainty. Currently, observations of HGPIN require conservative monitoring regimens without the justification of repeat biopsies [4], while AIP lesions warrant immediate follow-up, as such lesions may be indicative of unsampled IDCP and/or invasive prostate cancer [5].

The endosome lysosome biogenesis is integrally involved in all hallmarks of cancer, displaying altered biogenesis and function in prostate cancer biology [6–8]. A biomarker panel comprising Appl1, Sortilin, and Syndecan-1 has recently been used to define the complex biological changes contributing to prostate cancer pathogenesis and IDCP [9–11]. This study aimed to extend this research by evaluating the biomarker panel on prostatic precursor intraductal proliferative lesions to ascertain their suitability for inclusion within the current clinicopathological recommendations for guiding patient management.

## Methods

Patient tissue was sourced from the Kathleen Cuningham Foundation Consortium for research into Familial Breast cancer (kConFab). Serial, formalin-fixed paraffin-embedded, radical prostatectomy sections from 87 patients with prostatic adenocarcinoma (Table 1) were stained with haematoxylin and eosin (H&E) and labelled by IHC (the biomarker panel and PIN4 cocktail). Tissue sections were stained with routine Ehrlich’s H & E (Australian Biostain Pty Ltd., VIC, Australia), using the Leica ST5010 Autostainer XL (Leica Biosystems, VIC, Australia) automated staining platform. IHC labelling was performed on a Ventana BenchMark Ultra platform (Roche Diagnostics Pty Ltd., NSW, Australia). Briefly, epitope retrieval was performed in CC1 buffer (at 95 °C for 32 min for the biomarker panel, and 64 min for the 34βE12+P63/P504S; Roche, Australia). Tissue sections were then incubated with monoclonal antibodies to Appl1, Sortilin, or Syndecan-1, for 1 h at 36 °C, or with 34βE12+P63 for 32 min and P504S 16 min at 36 °C. Detection and visualisation were performed using the OptiView DAB Detection Kit (Roche, Australia). Tissue sections were counterstained with Gill’s haematoxylin (Roche, Australia) and mounted with D.P.X. mounting medium (Thermo Fisher Scientific, VIC, Australia). All tissue slides were scanned using a Zeiss Axio Scan.Z1 in brightfield mode, with a Plan-Achromat 20× objective (Zeiss, Germany). In total, tissue from 87 patients was stained by H&E and labelled by IHC, but one patient was excluded due to technical issues during immunolabelling. Intraductal lesions that were located within 2 mm of the adenocarcinoma were assessed.

**Table 1:** Patient characteristics

Patient characteristics						
Patients		86				
Patients with HGPIN or AIP		64				
Mean age (years)		61.8				
RP Grade Group	Total cases	No. patients with HGPIN or AIP	% Flat HGPIN (*n*)	% Tufting HGPIN (*n*)	% MP HGPIN (*n*)	% AIP (*n*)
1	13	9	56 (5)	78 (7)	67 (6)	33 (3)
2	36	26	58 (15)	81 (21)	73 (19)	23 (6)
3	18	17	59 (10)	65 (11)	41 (7)	71 (12)
4	2	1	100 (1)	100 (1)	100 (1)	100 (1)
5	11	9	67 (6)	56 (5)	78 (7)	67 (6)
NA	6	2	50 (1)	50 (1)	50 (1)	100 (2)
Total	86	64	59 (38)	72 (46)	64 (41)	47 (30)

*AIP* atypical intraepithelial neoplasia, *HGPIN* high-grade prostatic intraepithelial neoplasia, *MP* micropapillary, *RP* radical prostatectomy, *NA* RP Grade Group not available

## Results

### Intraductal non-IDCP lesions in prostate carcinoma

**Fig. 1:**
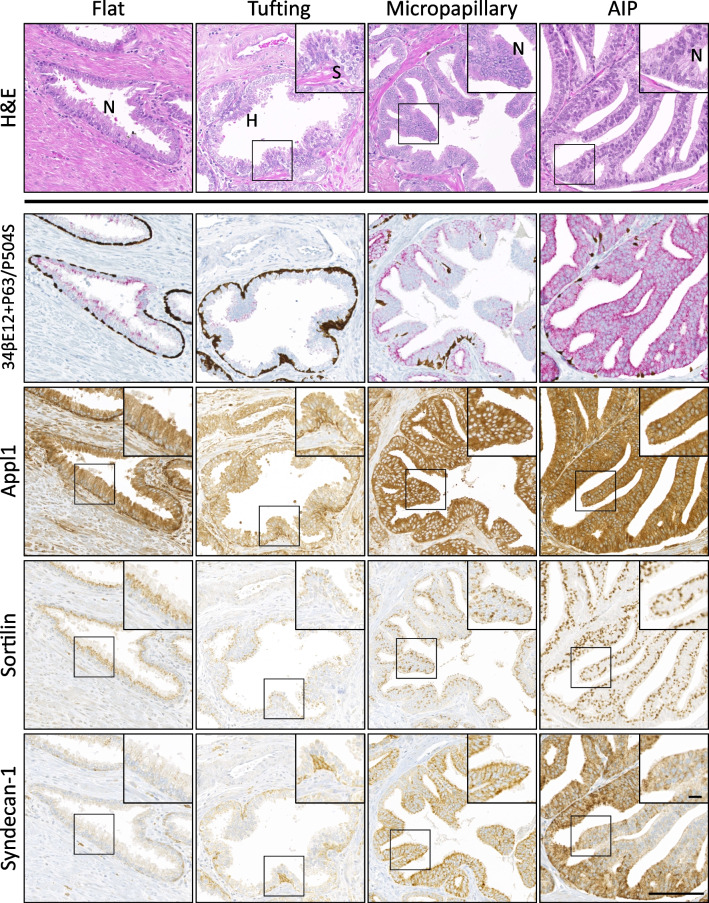
Biomarker panel expression in precursor lesions of prostate cancer. Representative regions of precursor HGPIN (flat, tufting, MP) and AIP lesions were stained with routine H&E (top row) or immunohistochemically labelled with antibodies against basal cell/AMACR cocktail (34βE12+P63/P504S; second row), Appl1 (third row), Sortilin (fourth row), and Syndecan-1 (fifth row). H, nuclear hyperchromasia; N, prominent nucleoli; S, stratification and crowding. Scale bar in image represents 100 μm (20 μm in inset)

Histological and IHC assessment of radical prostatectomy tissue samples enabled the identification of four non-IDCP intraductal lesions, which were found in 76.7% of cases. These were classified according to morphological features: nuclear enlargement/stratification/crowding and prominent nucleoli with or without nuclear hyperchromasia (Fig. 1). PIN4 immunolabelling often revealed an incomplete basal cell layer (34βE12+P63) and demonstrated variable P504S labelling of atypical secretory epithelial cells in HGPIN (flat, tufting, and MP) and AIP lesions (Fig. 1). The most common patterns of HGPIN were tufting and MP (72% and 64%, respectively), followed by flat (59%), and lesions assigned as AIP (47%) (Table 1). Furthermore, less than 23% of patients (*n* = 15) had one lesion alone, wherein flat, tufting, and MP HGPIN was found equally (6% patients). A total of 33% patients had two lesions occurring together, with the most common being tufting and MP HGPIN (11% patients). A total of 22% of patients displayed three lesions, the most common combination being flat, tufting, and MP HGPIN (9% patients), while another 22% of the patients had four lesions occurring together.

### Discrete biomarker expression pattern in prostate cancer precursor lesions and AIP

**Fig. 2:**
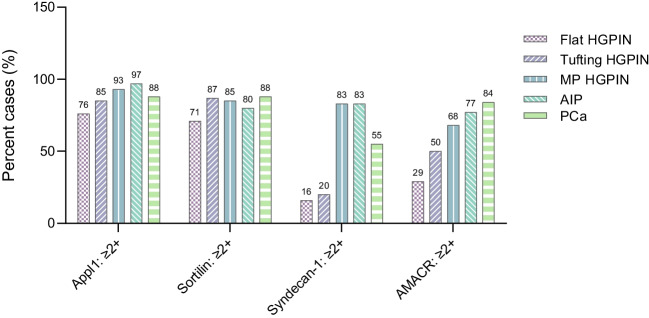
Expression pattern in atypical intraductal lesions of the prostate. Appl1/Sortilin/Syndecan-1 and AMACR expression in HGPIN, AIP, and prostatic adenocarcinoma. Intensity was scored 0–3+, and moderate-to-high (≥ 2) expression is shown

Appl1 labelled basal cells with high intensity, while atypical secretory epithelial cells in glands with HGPIN (flat, tufting, and MP) and AIP displayed moderate-to-high labelling intensity (≥ 76% cases, Fig. 2). Sortilin labelling was only detected in secretory cells, in a supranuclear location that was often diffuse in flat HGPIN (Fig. 1). Although Sortilin labelling was detected at a moderate-to-high intensity in all assessed patterns (≥ 71% cases), tufting HGPIN displayed a polarised distribution towards the periphery of the luminal proliferations. While Sortilin appeared disorganised in tufting HGPIN due to the pseudostratification of the cells, MP HGPIN and AIP displayed a highly organised distribution of Sortilin (Fig. 1). Syndecan-1 labelling intensity was moderate-to-strong in patterns with large epithelial proliferations (MP HGPIN and AIP; 83% cases), when compared to flat HGPIN or small luminal protrusions (tufting; ≤ 20% cases) (Fig. 2). The pattern of expression observed in MP HGPIN/AIP (Appl1 labelling with positive Sortilin and Syndecan-1) is similar to that in cribriform IDCP patterns [10]. The majority of the intraductal lesions were in the immediate vicinity of the invasive component (69%), while 31% were distant to the cancer, but within 2 mm distance. When the lesions were compared to the invasive component, the pattern of expression was similar, displaying moderate-to-high intensity of Appl1 and Sortilin in 88% of cases and moderate-to-high intensity of Syndecan-1 in 55% of cases. Notably, the majority of MP and AIP lesions (83%) had comparable or higher expression of Syndecan-1 than that expressed in the cancer component, versus levels observed in flat or tufting HGPIN lesions (20%) (Fig. 2).

## Discussion

The markers Appl1, Sortilin, and Syndecan-1, which are at critical control points in the endosome-lysosome system, form a biomarker panel that can accurately map the pathogenesis in prostate cancer and assist Gleason grading to enable more accurate prediction of biochemical and clinical recurrence in patients [9, 11]. More recently, this biomarker panel has shed light on the retrograde spread theory of IDCP, wherein cribriform patterns displayed strong Appl1, Sortilin, and Syndecan-1 labelling patterns, while solid IDCP architecture had high intensity Appl1 and Syndecan-1 labelling, but minimal Sortilin labelling [10].

Here, the expression pattern of the biomarker panel was assessed in atypical intraductal proliferative lesions in prostate cancer, revealing distinct patterns of expression with Appl1, Sortilin, and Syndecan-1, including (1) labelling of Appl1/Sortilin in flat/tufting HGPIN and (2) labelling of Appl1, Sortilin, and Syndecan-1 in MP HGPIN and AIP. The observation that the MP HGPIN and AIP have a similar pattern of expression is not surprising, since morphologically both patterns comprise large epithelial proliferations, suggesting that MP is an architecturally more advanced lesion than flat/tufting HGPIN. In accordance, the tufting/flat HGPIN pattern is associated with a significantly lower risk of cancer upon follow-up, when compared to MP HGPIN and cribriform type HGPIN (a lesion now classified as either AIP or diagnosed as IDCP if there is sufficient atypia) [12].

While lesions assigned the term AIP share similar ERG+/PTEN status with IDCP, this expression profile is not observed in HGPIN lesions [13, 14], but these studies did not segregate HGPIN patterns. In this study, the segregation of patterns revealed that MP HGPIN lesions share a similar Appl1/Sortilin/Syndecan-1 profile to AIP/IDCP lesions [10], raising two important considerations. Firstly, according to current clinicopathological recommendations, AIP requires immediate repeat biopsy as this may be indicative of unsampled IDCP, but a similar consideration may now be warranted for MP HGPIN lesions displaying this labelling pattern. Secondly, some cases of AIP cannot be definitively diagnosed as IDCP for a variety of reasons, including incomplete sampling by needle biopsy. However, this study shows that some AIP lesions display the same Appl1/Sortilin/Syndecan-1 labelling pattern that is observed in IDCP (loose/dense cribriform) [10]. This suggests that the panel of biomarkers has utility in identifying IDCP in lesions previous termed AIP.

Management of patients with precursor lesions of prostate cancer is dependent on the accurate distinction between HGPIN patterns and those assigned AIP. The Appl1, Sortilin, and Syndecan-1 biomarker panel can depict either early atypical changes (flat/tufting HGPIN) or more advanced atypical changes (MP HGPIN/AIP). Furthermore, the biomarker panel may be used as a tool to overcome the diagnostic uncertainty surrounding AIP by supporting a definitive diagnosis of IDCP for such lesions displaying the same labelling pattern as cribriform IDCP. The results from this study shed light on precursor lesions of prostate cancer and present a novel panel of biomarkers that can be used to guide patient management.

## Data Availability

The data that support the findings of this study are not openly available due to reasons of sensitivity and are available from the corresponding author upon reasonable request.
