# Publisher Correction to: Distinct patterns of biomarker expression for atypical intraductal proliferations in prostate cancer

**DOI:** 10.1007/s00428-023-03666-8

**Published:** 2023-09-29

**Authors:** Carmela Martini, Jessica M. Logan, Alexandra Sorvina, Sarita Prabhakaran, Benjamin S-Y. Ung, Ian R. D. Johnson, Shane M. Hickey, Robert D. Brooks, kConFab Consortium, Maria C. Caruso, Sonja Klebe, Litsa Karageorgos, John J. O’Leary, Brett Delahunt, Hemamali Samaratunga, Douglas A. Brooks

**Affiliations:** 1 Clinical and Health Sciences, University of South Australia, Adelaide, SA Australia; 2 Department of Anatomical Pathology, College of Medicine and Public Health, Flinders University, Adelaide, SA Australia; 3 Department of Surgical Pathology, SA Pathology at Flinders Medical Centre, Adelaide, SA Australia; 4 Department of Histopathology, Trinity College Dublin, Dublin, Ireland; 5 Malaghan Institute for Medical Research, Wellington, New Zealand; 6 Aquesta Uropathology and the University of Queensland, Brisbane, Brisbane, Qld Australia

**Publisher Correction to: Virchows Archiv**

**https://doi.org/10.1007/s00428-023-03643-1**

The publisher regrets that the original published version of the above article contained incomplete authors.

The following co-authors should have been among the main authors; however, they were listed as collaborators under the kConFab Consortium.

**Maria C. Caruso, Sonja Klebe, Litsa Karageorgos, John J. O’Leary, Brett Delahunt, Hemamali Samaratunga, Douglas A. Brooks**

The original article has been corrected.

